# Synergistic Effects of Sanguinarine and *Achyranthes bidentata* Polysaccharides on Growth and Immunity in Yellow-Feathered Broilers

**DOI:** 10.3390/vetsci13010036

**Published:** 2026-01-01

**Authors:** Xiaolong Wang, Zhuying Liu, Longteng Ma, Wenbin Chen

**Affiliations:** 1 College of Biological and Environmental Engineering, Yueyang Vocational Technology College, Yueyang 414000, China; 13627488848@163.com; 2 College of Animal Science and Technology, Hunan Biological and Electromechanical Polytechnic, Changsha 410128, China; 3 Animal Nutritional Genome and Germplasm Innovation Research Center, College of Animal Science and Technology, Hunan Agricultural University, Changsha 410128, China; malongteng2021@163.com; 4 Department of Animal Science and Technology, Zhong kai University of Agriculture and Engineering, Guangzhou 510000, China

**Keywords:** sanguinarine, *Achyranthes bidentata* polysaccharides, lymphocyte proliferation, broiler chicken

## Abstract

The search for natural alternatives to in-feed antibiotics is a central focus in sustainable poultry production. This study investigated the individual and combined effects of Sanguinarine (SA) and *Achyranthes bidentata* polysaccharides (ABPS), two bioactive compounds derived from Chinese herbs, on the growth performance and immune function of yellow-feathered broilers. A total of 1728 one-day-old female broilers were randomly allocated to 36 treatment groups in a 6 × 6 factorial arrangement, with factors being six levels of SA (0, 0.4, 0.5, 0.6, 0.7, and 0.75 mg/kg) and six levels of ABPS (0, 200, 300, 400, 500, and 600 mg/kg). Over an 8-week period, growth performance indicators, including average daily gain (ADG), average daily feed intake (ADFI), and feed conversion ratio (FCR), were monitored. Immune status was assessed through relative weights of immune organs (thymus, bursa of Fabricius, spleen) and lymphocyte proliferation at 4 and 8 weeks of age. The results demonstrated that SA supplementation significantly improved ADG during the initial 0–2-week period, while its effects on ADFI and FCR varied across different growth stages. ABPS supplementation was most effective in reducing FCR throughout the experiment, with the optimal effect observed at 500 mg/kg. Regarding immune parameters, SA at 0.7 mg/kg yielded the highest relative immune organ weights and lymphocyte proliferation at 4 weeks, whereas 0.5 mg/kg was most effective for lymphocyte proliferation at 8 weeks. ABPS at 500 mg/kg consistently promoted immune organ development and lymphocyte proliferation. A significant synergistic interaction between SA and ABPS was identified for several key outcomes. This interaction influenced ADG and ADFI at 8 weeks of age, relative thymus weight at 4 weeks, and lymphocyte proliferation at both 4 and 8 weeks. Notably, the combination of SA at 0.5 mg/kg and ABPS at 300 mg/kg (A_3_B_3_) produced a marked enhancement in lymphocyte proliferation. In conclusion, dietary supplementation with SA, ABPS, or their combination can modulate growth performance and enhance immune function in yellow-feathered broilers. The effects are dose-dependent and can be synergistic, with the combination of 0.5 mg/kg SA and 300 mg/kg ABPS showing particular promise for boosting cell-mediated immunity. These findings support the potential of SA and ABPS as beneficial natural feed additives for antibiotic-free poultry production.

## Simple Summary

The overuse of antibiotics in poultry farming is a global concern, driving the search for effective natural alternatives. This study explores the potential of two plant-based compounds—sanguinarine (SA) and *Achyranthes bidentata* polysaccharides (ABPS)—as feed additives for yellow-feathered broilers. We investigated how these supplements, when used alone or together, affect the birds’ growth and immune health. Our findings show that both SA and ABPS can improve weight gain and feed efficiency at specific stages of growth. More importantly, we discovered that combining them creates a synergistic effect, meaning their joint action is more powerful than the sum of their individual effects. A key finding is that a combination of 0.5 mg/kg of SA and 300 mg/kg of ABPS was particularly effective in boosting the birds’ immune responses. This research provides strong evidence that SA and ABPS can be used as a natural and effective strategy to promote health and productivity in broiler chickens, contributing to sustainable and antibiotic-free poultry production.

## 1. Introduction

**Figure 1:**
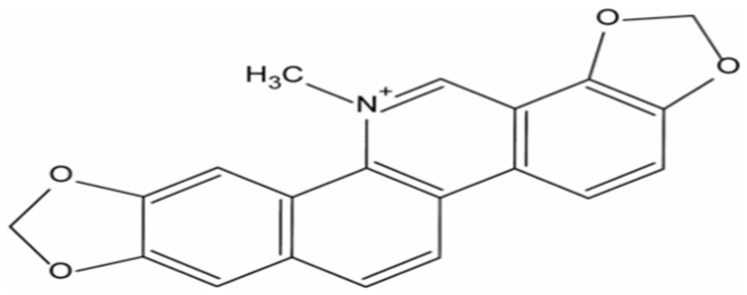
The structures of Sanguinarine (SA, C_20_H_14_NO_4_) [5].

Considerable controversy still surrounds the utilization of antibiotics for promoting poultry growth intended for human consumption [1,2]. Consequently, extensive research has been conducted to explore natural compounds as alternatives to reduce antibiotic usage. Recent studies have demonstrated the immunomodulatory properties of various Chinese herbal medicines and their constituents in growing pigs and broilers, including Angelica sinensis, polysaccharides, and Sanguinarine (SA, C_20_H_14_NO_4_) [3,4]. However, the majority of existing research focuses on the effects of these compounds in isolation, with limited exploration of their potential synergistic effects or their application in specific poultry breeds such as yellow-feathered broilers. Sanguinarine is a quaternary benzophenanthridine alkaloid derived from phenylalanine in plants belonging to the Papaveraceae family (Figure 1) [5,6]. SA exhibits a diverse range of biological activities encompassing antifungal, anti-inflammatory, antimicrobial, analgesic, and anticancer properties in humans as well as broilers and pigs [7,8,9,10]. The inclusion of SA in poultry diets has been employed to enhance feed intake and stimulate growth [10,11]. It is recommended that farm animals be administered Sangrovit at a dosage of 20–100 ppm (containing 1.5% SA with extract powder) in their feed [12]. However, due to the shorter length of midgut and hindgut in poultry, it is necessary to adjust the recommended dosage according to the specific needs of broilers [13]. Notably, while studies have shown that supplementation with Sangrovit (50 ppm for 1–21 days and 25 ppm for 22–42 days) improves body weight at three weeks of age in broilers [11], its effects on immune modulation remain underexplored.

**Figure 2:**
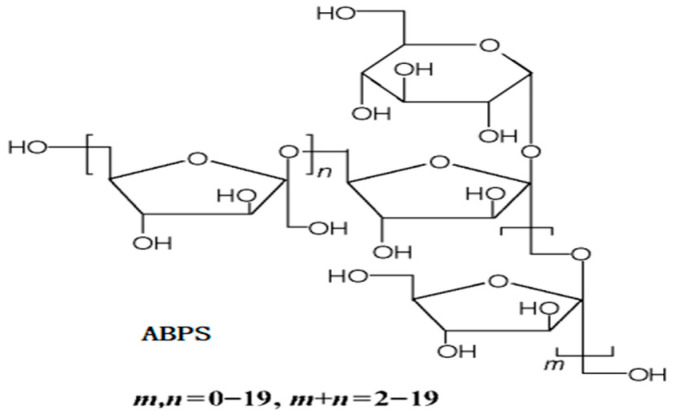
The structures of *Achyranthes bidentata* Polysaccharides (ABPS) [15].

Polysaccharides derived from *Achyranthes bidentata* (ABPS) have been extensively utilized in traditional medicine across China, Japan, and Korea for their preventive and therapeutic properties against a wide range of human ailments [14]. The ABPS consists of fructose and glucose residues, with a molecular weight ranging from 1000 to 1400 Da (Figure 2) [15]. At an appropriate concentration, ABPS exhibits a broad spectrum of immunomodulatory effects, including the stimulation of splenocyte proliferation and activation of macrophages, NK cells, T cells, and B cells. The addition of ABPS has been reported to enhance the in vitro maturation and differentiation of dendritic cells by increasing the population of myeloid (CD11c^+^ CD11b^+^) and plasmacytoid dendritic cells (CD11c^+^ CD45R^+^/B220^+^), as well as regulating the expression of MHC-II, CD86, particularly Toll-like receptor 9 by CD11c^+^ dendritic cells [16,17]. It is recommended to supplement swine diets with 200mg to 500 mg ABPS for improved immune function, feed intake, and growth [18,19,20]. Despite these promising findings, limited studies have evaluated the effects of ABPS on poultry immune responses and growth performance, particularly in combination with other bioactive compounds like SA.

In previous studies, we observed that the inclusion of ABPS in pig’s diet resulted in increased feed intake, improved health status, and enhanced growth [19,21]. Despite the well-documented individual benefits of SA and ABPS, two critical knowledge gaps remain. First, the existing literature predominantly focuses on the effects of each compound in isolation. There is a notable lack of research exploring their potential synergistic effects when used in combination, which could potentially yield efficacy superior to the sum of their individual actions. Second, while both are proposed as antibiotic alternatives, comprehensive dose–response studies—essential for determining their optimal application—are scarce, particularly in specific poultry breeds like yellow-feathered broilers which have distinct nutritional requirements. It is important to clarify that the primary objective of this study is not to conduct a direct head-to-head comparison with ATB, but rather to systematically investigate the individual and interactive effects of SA and ABPS. This foundational work is a necessary step to establish their synergistic potential and optimal dosing regimens, which will provide a solid basis for future studies designed to benchmark these natural additives against the industry standard of ATB.

Therefore, this study was designed to fill these gaps by employing a comprehensive factorial design. We aim to: (1) evaluate the individual effects of a wide range of SA and ABPS doses on the growth performance and immune function of yellow-feathered broilers; and (2) critically assess whether a synergistic interaction exists between SA and ABPS that could enhance their efficacy. By identifying the most effective single doses and combinations, this research aims to provide robust scientific evidence for developing effective, multi-component antibiotic-alternative strategies in sustainable poultry production.

## 2. Materials and Methods

This study was conducted to evaluate the impact of SA, ABPS, and their combinations on key productivity parameters in yellow-feathered broilers. The trial followed a two-phase feeding program, consisting of a starter phase (days 1–28) and a finisher phase (days 29–56), to precisely meet the changing nutritional needs of the birds. Basal diets for both phases were formulated based on the nutrient requirements outlined in China’s NY/T 33-2004 [22] guidelines and the NRC (1994) [23] (Table 1). The entire experimental protocol was reviewed and approved by the Institutional Animal Care and Use Committee of Hunan Biological and Electromechanical Polytechnic.

### 2.1. Preparation of ABPS

The *Achyranthes bidentata* polysaccharide (ABPS) was provided by the National & Local Joint Engineering Research Center of Veterinary Herbal Medicine Resources and Veterinary Medicine Innovation at Hunan Agricultural University, Changsha, China. The extract consisted of D-fructose and D-glucose in a molar ratio of 8:1, as determined by HPLC analysis conducted at the Shanghai Institute of Chemical Physics, Chinese Academy of Sciences, China. The final product exhibited a purity of 98% ABPS, measured using vitriol anthrone with anhydrous glucose as the standard control. Gel filtration method revealed that the relative molecular mass of ABPS ranged from 1300 to 1400 Da. This purified extract powder was utilized for conducting this trial.

### 2.2. Preparation of SA

The extraction of sanguinarine from Macleaya cordata was conducted at the National & Local Joint Engineering Research Center of Veterinary Herbal Medicine Resources and Veterinary Medicine Innovation at Hunan Agricultural University, Changsha, China, using ethanol as previously described [24]. Subsequently, the extracted sanguinarine was purified through silica gel column chromatography, yielding a purity level of 99% as determined by HPLC analysis (First, the plant material is processed using methods such as ultrasonic-assisted extraction or enzymatic pretreatment to obtain a crude extract of sanguinarine. Subsequently, a Cosmosil C18-R-Ⅱ column (250 mm × 4.6 mm, 5 μm) is selected to ensure optimal separation efficiency. The mobile phase consists of acetonitrile and 0.2% acetic acid solution in a ratio of 25:75 (*v*/*v*), which balances good separation performance with enhanced analytical speed. The detection wavelength is set at 270 nm to achieve optimal absorbance signals. During the purification process, high-purity extraction of sanguinarine is achieved by optimizing parameters such as sample concentration (6.0 mg/mL), sample pH (5.5), and loading volume (25 mL). Finally, the purified sanguinarine is verified for its purity and structural integrity using methods including HPLC, UV spectrophotometry, mass spectrometry, and nuclear magnetic resonance spectroscopy). The resulting extract powder was utilized for the present study.

### 2.3. Diets and Experimental Design

The experimental procedures were approved by the Institutional Animal Care and Use Committee of Hunan Agricultural University. A total of 1728 day-old yellow female broiler chickens (average BW = 30.3 g) were randomly allotted to a 6 × 6 factorial arrangement of 36 dietary treatments. Each treatment was replicated in 6 pens (cages), with each pen containing 8 birds, resulting in a total of 216 pens. The pen was the experimental unit for the analysis of growth performance. For the analysis of blood parameters and tissue samples, one bird was selected from each pen, resulting in 6 individual birds (the experimental unit for these endpoints) being sampled per treatment. Pens were arranged in a three-tier battery cage system. The vertical tier was used as a blocking factor. The 36 dietary treatments were randomly assigned to pens within each tier, with the constraint that each treatment was represented equally across all three tiers to form a complete block. This resulted in each treatment having two replicate pens located on each of the top, middle, and bottom tiers. Birds were fed corn-soybean meal diets without the use of growth promoters or anticoccidials and received coccidiosis vaccination upon placement. A complete random arrangement of treatments using a 2-factorial design with six levels was employed for the trial. Two Chinese herb extracts, namely SA and ABPS, were utilized in this study, each having six different concentrations as detailed in Table 2. Broilers from each treatment group were provided with commercial broiler starter diets (0–28 days) and finisher diets (29–56 days), supplemented with the corresponding concentration of extract as specified in Table 1 and Table 2.

The body weight of each individual bird within every replicate was measured separately at 0, 2, 4, 6, and 8 weeks of age to ensure precise data collection. Daily records of mortality and feed intake were maintained for each cage. Using the collected data, average daily feed intake (ADFI), average daily body weight gain (ADG), and feed conversion ratio (FCR) were calculated for each replicate.

### 2.4. Sampling Collection

Six birds were randomly selected from each group (one bird per cage) and subjected to a 12 h fasting period prior to sacrifice. The final live weight of each chicken was determined upon euthanasia (Euthanasia System: We employed the ZL-006 system, Isoflurane was administered at a concentration of 1–4% for anesthesia until the animal lost consciousness, followed by switching to carbon dioxide for euthanasia). Euthanized chickens were used for the collection of immune organs, including thymus, spleen, and bursa, with subsequent evaluation of organ weights. Relative organ weight was calculated as the ratio of organ weight (in grams) to body weight (in kilograms). All experimental procedures were approved by the Institutional Animal Care and Use Committee of Hunan Biological and Electromechanical Polytechnic (Changsha, China) (Protocol No. 2020-09). At 4 and 8 weeks of age, six birds were randomly chosen from each group (one bird per cage) and underwent a 12 h fasting period before sacrifice. A blood sample volume of five mL per bird was collected via venipuncture from the wing vein.

### 2.5. Lymphocyte Proliferation Assay

Whole blood lymphocyte proliferation assay was performed as previously described [25]. Fresh heparinized blood was briefly subjected to Percoll gradient centrifugation for the isolation of lymphocytes. The isolated lymphocytes were then suspended in RPMI 1640 medium (Sigma-Aldrich, Inc., St. Louis, MO, USA) supplemented with 100 U/mL penicillin, 100 μg/mL streptomycin, and 2 mM L-Gln (complete media), and further supplemented with 10% heat-inactivated fetal bovine serum (Qian Yuan Hao Biological Co., Ltd., Beijing, China). Subsequently, a volume of 100 μL of the lymphocyte suspension along with phytohemagglutinin (PHA, Sigma-Aldrich Inc.) was added to each well of a 96-well microtiter plate to achieve a final concentration of PHA at 20 μg/mL [26]. No lymphocytes were added to the positive control wells, while lymphocytes without PHA addition were used as negative controls. Following a 48 h incubation at 37 °C and 5% CO_2_ in an incubator, each well was supplemented with 10 μL of MTT (3-(4,5-dimethylthiazolyl)-2,5-diphenyl tetrazolium bromide) solution (5 mg/mL, Sigma-Aldrich Inc.) and further incubated for 4 h. Subsequently, the plates were centrifuged at room temperature for 10 min at a speed of 1000× *g*. The supernatant was carefully aspirated and replaced with 100 μL of DMSO per well. After shaking the plates for complete crystal dissolution over a period of 5 min, cell absorbance was measured using an automated microplate analyzer (MQX200, Bio-Tek Instruments Inc., Winooski, VT, USA) at a wavelength of 570 nm (A570 value), serving as an indicator of lymphocyte proliferation.

### 2.6. Statistical Analysis

The data were analyzed using one-way and two-way ANOVA for single-factor and two-factor designs, respectively, with SPSS (SPSS Statistics 20). Multiple comparisons between the six groups in a single-factor design were conducted using the Duncan method to determine significant differences. General linear modules in SPSS 20.0 software were employed to evaluate the interaction between treatments in a two-factor design, and mean differences among different groups were assessed using Duncan’s multiple range tests. Significance was defined as a *p*-value ≤ 0.05.

## 3. Result

### 3.1. Growth Performance

**Figure 3:**
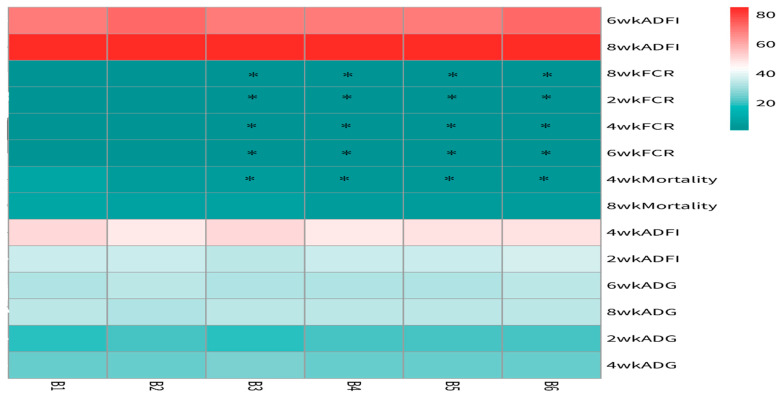
Effect of ABPS on growth performance in yellow broilers on different ages. * Means a significant difference (*p* < 0.05). B factor was *Achyranthes bidentata* polysaccharides (ABPS), with six levels set as B_1_ 0 mg/kg, B_2_ 200 mg/kg, B_3_ 300 mg/kg, B_4_ 400 mg/kg, B_5_ 500 mg/kg, and B_6_ 600 mg/kg; *n* = 36.

The statistical analysis results from Figure 3 indicate that the addition of *Achyranthes bidentata* polysaccharides (ABPS) at 200 mg/kg (B_2_) and 600 mg/kg (B_6_) significantly improved the average daily gain (ADG) and feed intake of yellow-feathered broilers during the 0–6 week period (*p* = 0.001), while no significant effects were observed during the 0–2 week, 0–4 week, or 0–8 week periods. The addition of different doses of ABPS effectively reduced the feed conversion ratio (FCR) during the experimental period (*p* < 0.001), with the effect being most pronounced at 500 mg/kg (B_5_). Furthermore, ABPS supplementation at 300 mg/kg, 400 mg/kg, 500 mg/kg, and 600 mg/kg effectively reduced the mortality rate of yellow-feathered broilers during the 0–4-week period.

**Figure 4:**
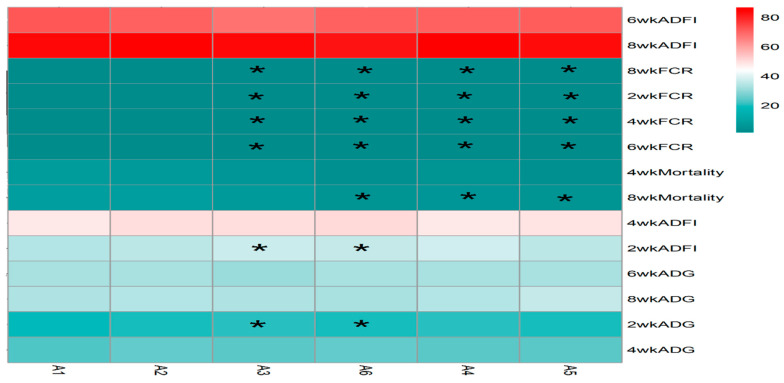
Effect of SA on growth performance in yellow broilers at different ages. * Means a significant difference (*p* < 0.05). A factor was sanguinarine (SA), with six levels were set as A_1_ 0 mg/kg, A_2_ 0.4 mg/kg, A_3_ 0.5 mg/kg, A_4_ 0.6 mg/kg, and A_5_ 0.7 mg/kg, A_6_ 0.75 mg/kg; *n* = 36.

The statistical analysis results from Figure 4 indicate that the addition of sanguinarine at 0.4 mg/kg (A_2_), 0.7 mg/kg (A_5_), and 0.75 mg/kg (A_6_) had no significant effects on the average daily gain (ADG) and feed intake of yellow-feathered broilers during the 0–2-week period. However, the addition of sanguinarine at 0.5 mg/kg and 0.6 mg/kg significantly improved the ADG and feed intake during the 0–2-week period (*p* = 0.006), while no significant effects were observed in other stages. Sanguinarine supplementation levels of 0.5 mg/kg, 0.6 mg/kg, 0.7 mg/kg, and 0.75 mg/kg effectively reduced the feed conversion ratio (FCR) during the experimental period (*p* < 0.05), although there were no significant differences among the sanguinarine groups. Additionally, sanguinarine supplementation at 0.6 mg/kg, 0.7 mg/kg, and 0.75 mg/kg effectively reduced the mortality rate of yellow-feathered broilers during the experimental period.

**Figure 5:**
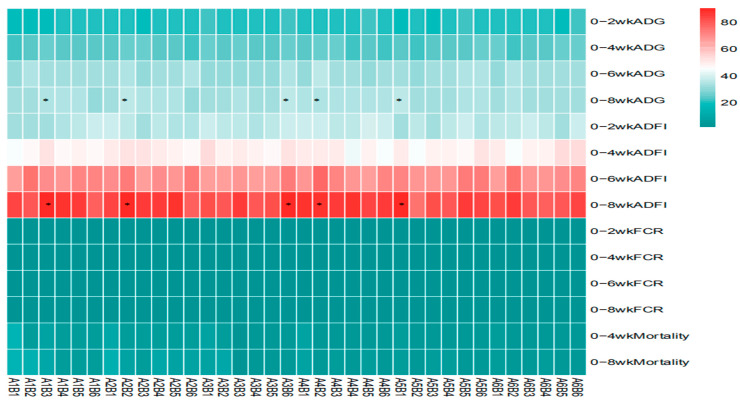
Effect of ABPS and SA combinations on growth performance in yellow broilers on different ages. * Means a significant difference (*p* < 0.05). A factor was sanguinarine (SA), with six levels were set as A_1_ 0 mg/kg, A_2_ 0.4 mg/kg, A_3_ 0.5 mg/kg, A_4_ 0.6 mg/kg, and A_5_ 0.7 mg/kg, A_6_ 0.75 mg/kg; B factor was *Achyranthes bidentata* polysaccharides (ABPS), with six levels set as B_1_ 0 mg/kg, B_2_ 200 mg/kg, B_3_ 300 mg/kg, B_4_ 400 mg/kg, B_5_ 500 mg/kg, and B_6_ 600 mg/kg. *n* = 36.

The statistical analysis results from Table 3 and Figure 5 show that sanguinarine significantly affected the average daily gain (ADG) of yellow-feathered broilers during the 0–2-week period (*p* = 0.006), while no significant effects were observed in other age stages (*p* > 0.05). *Achyranthes bidentata* polysaccharides (ABPS) significantly influenced the ADG during the 0–6-week period (*p* = 0.001), with no significant effects in other age stages (*p* > 0.05). There was no significant interaction between sanguinarine and ABPS on the ADG of broilers before 6 weeks of age (*p* > 0.05); however, a significant interaction was observed for the 0–8-week ADG (*p* = 0.002). The combinations A_1_B_3_, A_1_B_4_, A_2_B_2_, A_2_B_5_, A_3_B_6_, A_4_B_2_, A_4_B_4_, and A_5_B_1_ showed significant effects. Sanguinarine significantly affected the feed intake of yellow-feathered broilers during the 0–2 week, 0–6 week, and 0–8-week periods (*p* < 0.05), while no significant effects were observed during the 0–4 week period (*p* = 0.15). ABPS significantly influenced the average feed intake of broilers during the 0–6-week period (*p* = 0.001), with no significant effects in other age stages (*p* > 0.05). No significant interaction was observed between sanguinarine and ABPS on feed intake before 6 weeks of age (*p* > 0.05); a significant interaction was observed for the 0–8-week average feed intake (*p* = 0.03), with combinations A_1_B_3_, A_2_B_2_, A_3_B_6_, A_4_B_2_, and A_5_B_1_ showing significant effects. Sanguinarine significantly reduced the feed conversion ratio (FCR) of yellow-feathered broilers (*p* < 0.05), and ABPS also had a significant impact on FCR (*p* < 0.05). However, no significant interaction was observed between sanguinarine and ABPS on FCR (*p* > 0.05). ABPS significantly reduced the mortality rate of broilers during the 0–4-week period, and sanguinarine significantly reduced the mortality rate during the 0–8-week period. There was no significant interaction between sanguinarine and ABPS on the mortality rate of yellow-feathered broilers (*p* > 0.05).

### 3.2. Relative Weight of Immune Organs

**Figure 6:**
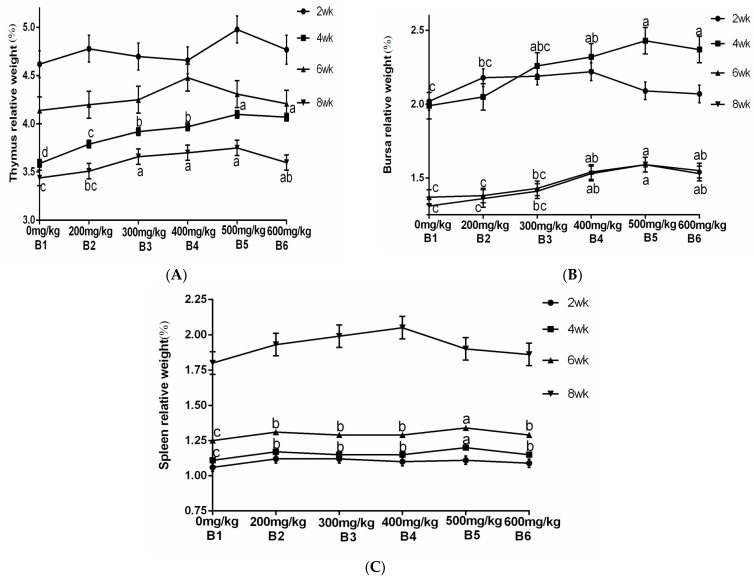
Effect of ABPS on the relative weight of immune organs in yellow broilers on different ages. (**A**) Thymus relative weight; (**B**) Bursa relative weight; (**C**) Spleen relative weight. Values in the same line without any letter or with the same letter superscripts indicate no significant difference (*p* > 0.05), while those with different small letter superscripts indicate a significant difference (*p* < 0.05). The factor B was ABPS, consisting of six levels: B_1_ 0 mg/kg, B_2_ 200 mg/kg, B_3_ 300 mg/kg, B_4_ 400 mg/kg, B_5_ 500 mg/kg, and B_6_ 600 mg/kg. *n* = 36.

**Figure 7:**
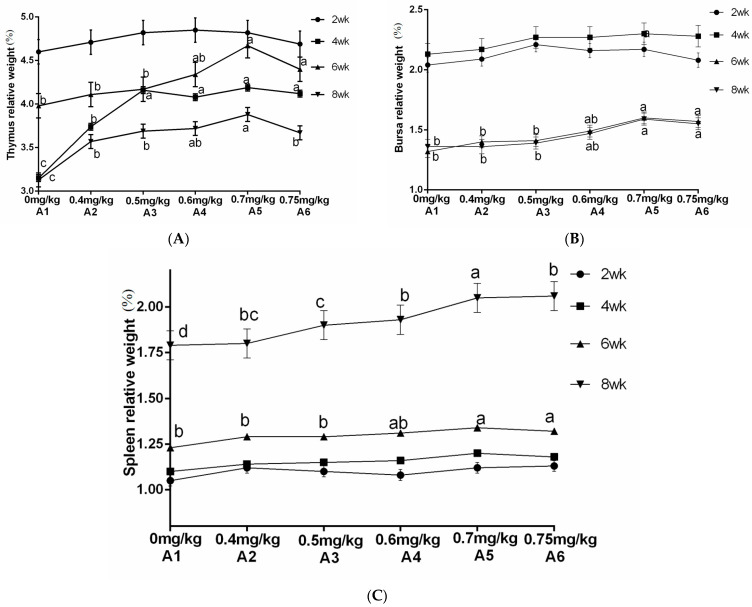
Effect of SA on the relative weight of immune organs in yellow broilers on different ages. The relative weights of the thymus (**A**), bursa (**B**), and spleen (**C**) were assessed. Values in the same line without any letter or with the same letter superscripts indicate no significant difference (*p* > 0.05), while values with different small letter superscripts indicate a significant difference (*p* < 0.05). Sanguinarine was considered as a factor and six levels were tested: A_1_ 0 mg/kg, A_2_ 0.4 mg/kg, A_3_ 0.5 mg/kg, A_4_ 0.6 mg/kg, and A_5_ 0.7 mg/kg, A_6_ 0.75 mg/kg. *n* = 36.

The results from Table 4 and Figure 6 demonstrated a significant increase in the relative weight of the thymus at 4 and 8 weeks of age when supplemented with ABPS. Additionally, there was a significant increase in the relative weight of the spleen at 4 and 6 weeks of age, while for bursa, a significant increase was observed after reaching 4 weeks of age. Notably, the highest relative organ weights were observed with ABPS treatment at a dosage of 500 mg/kg (B_5_). As shown in Table 4 and Figure 7, supplementation with SA significantly increased the relative weight of the thymus after reaching two weeks of age. Furthermore, there was a significant increase in both spleen and bursa weights after reaching four weeks of age among broilers receiving SA supplementation. Remarkably, the highest relative organ weights were observed with SA treatment at a dosage of 0.7 mg/kg (A_5_).

**Figure 8:**
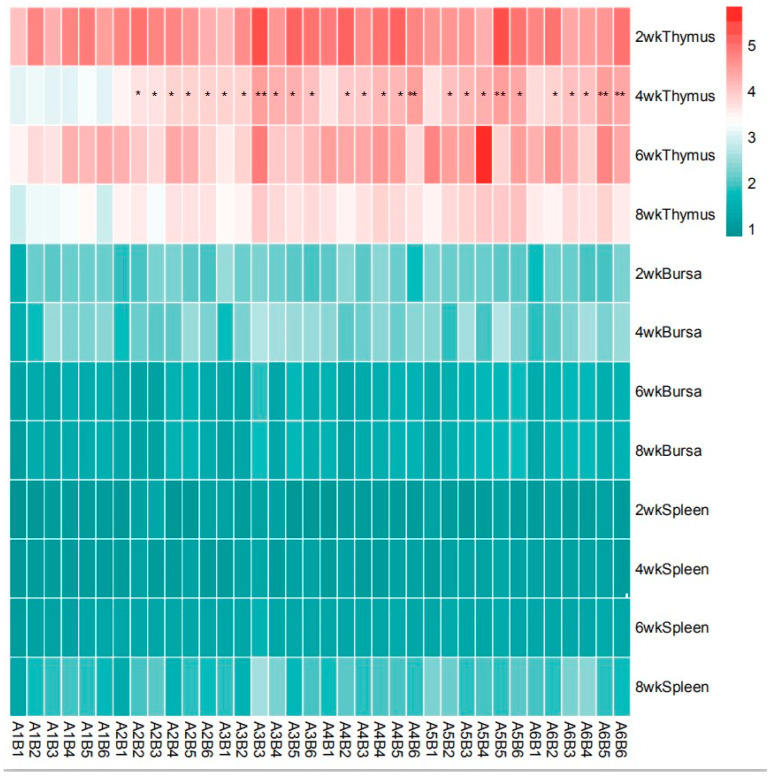
Effect of ABPS and SA combination on the relative weight of immune organs in yellow broilers on different age. * Means a significant difference (*p* < 0.05), ** Means an extremely significant difference (*p* < 0.01). A factor was sanguinarine (SA), with six levels were set as A_1_ 0 mg/kg, A_2_ 0.4 mg/kg, A_3_ 0.5 mg/kg, A_4_ 0.6 mg/kg, and A_5_ 0.7 mg/kg, A_6_ 0.75 mg/kg; B factor was *Achyranthes bidentata* polysaccharides (ABPS), with six levels set as B_1_ 0 mg/kg, B_2_ 200 mg/kg, B_3_ 300 mg/kg, B_4_ 400 mg/kg, B_5_ 500 mg/kg, and B_6_ 600 mg/kg. *n* = 36.

As presented in Table 4 and Figure 8, there was no significant interaction observed between SA and ABPS on the relative weight of spleen and bursa throughout the entire experimental period in yellow-feathered broilers (*p* > 0.05). However, a significant interaction was found between SA and ABPS on the relative weight of thymus at 4 weeks of age, particularly in A_3_B_3_, A_4_B_6_, A_3_B_6_, A_5_B_5_, A_6_B_5_, and A_6_B_6_ treatments (*p* < 0.05).

### 3.3. The Lymphocyte Proliferation of Broiler

**Figure 9:**
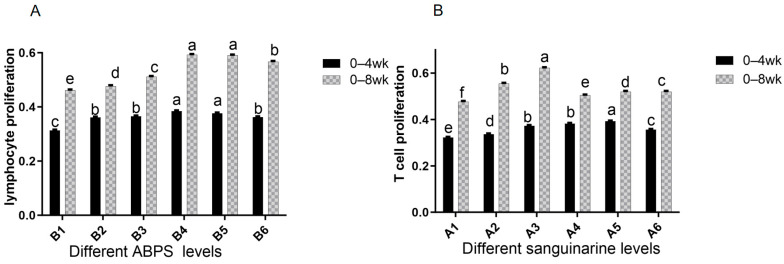
Effect of SA or ABPS on lymphocyte proliferation in yellow-feathered broilers. (**A**) Effects SA on the lymphocyte proliferation of broilers in yellow broilers on different ages; (**B**) Effects ABPS on the lymphocyte proliferation of broilers in yellow broilers on different ages; Each mean represents 36 replicates. OD values are calculated as optical density at 570 nm. *n* = 36. In the same row, values without any superscripts or with identical letter superscripts indicate no statistically significant difference (*p* > 0.05), while those with different lowercase letter superscripts indicate a significant difference (*p* < 0.05). One factor examined was sanguinarine, which had six levels: A_1_ 0 mg/kg, A_2_ 0.4 mg/kg, A_3_ 0.5 mg/kg, A_4_ 0.6 mg/kg, and A_5_ 0.7 mg/kg, as well as A_6_ 0.75 mg/kg; Another factor investigated was *Achyranthes bidentata* polysaccharide (ABPS), also with six levels: B_1_ 0 mg/kg, B_2_ 200 mg/kg, B_3_ 300 mg/kg, B_4_ 400 mg/kg, B_5_ 500 mg/kg, and B_6_ 600 mg/kg.

**Figure 10:**
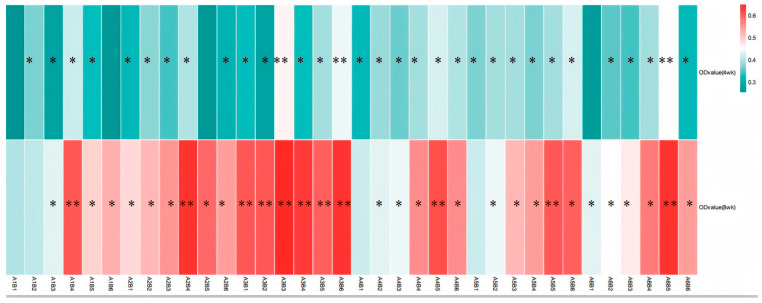
Effect of ABPS and SA combination on the lymphocyte proliferation of broilers in yellow broilers. * Means a significant difference (*p* < 0.05), ** Means an extremely significant difference (*p* < 0.01). A factor was sanguinarine (SA), with six levels were set as A_1_ 0 mg/kg, A_2_ 0.4 mg/kg, A_3_ 0.5 mg/kg, A_4_ 0.6 mg/kg, and A_5_ 0.7 mg/kg, A_6_ 0.75 mg/kg; B factor was *Achyranthes bidentata* polysaccharides (ABPS), with six levels set as B_1_ 0 mg/kg, B_2_ 200 mg/kg, B_3_ 300 mg/kg, B_4_ 400 mg/kg, B_5_ 500 mg/kg, and B_6_ 600 mg/kg. The heat map shows the combined effects of SA (A factor) and ABPS (B factor) on lymphocyte proliferation in broilers at 4 and 8 weeks. The color intensity represents the OD values measured at 570 nm, with darker red indicating higher values. Statistical significance (*p* < 0.05) is denoted by asterisks (*). Notably, the A_3_B_3_ group (0.5 mg/kg SA and 300 mg/kg ABPS) demonstrated the highest OD values at both 4 and 8 weeks, as reflected by the darkest red shading. This aligns with the statistical analyses shown in Table 4 and Figure 9, emphasizing the sustained immune-enhancing effects of A_3_B_3_. *n* = 36.

The results from Table 4 and Figure 9 demonstrate that dietary supplementation of SA and ABPS can enhance lymphocyte proliferation in broilers. Specifically, the addition of SA and ABPS significantly increased lymphocyte proliferation at both 4 and 8 weeks of age. At a dosage of 0.7 mg/kg (A_5_), SA showed the highest level of lymphocyte proliferation at week 4, while at a dosage of 0.5 mg/kg (A_3_), it was most effective at week 8 (Figure 9A). Similarly, ABPS supplementation at a dosage of 500 mg/kg (B_5_) resulted in the highest levels of lymphocyte proliferation at both time points (Figure 9B). Furthermore, our findings from Figure 9 and Figure 10 indicate a significant interaction between SA and ABPS on broiler lymphocyte proliferation during weeks four to eight (*p* < 0.05). Specifically, the A_3_B_3_ group (0.5 mg/kg SA and 300 mg/kg ABPS) consistently demonstrated the highest lymphocyte proliferation levels across both week 4 and week 8. In Figure 10, this is visually represented by the darker red shading corresponding to the A_3_B_3_ group, which indicates the highest observed OD values. These results are statistically validated in Table 4 and Figure 9, where the A_3_B_3_ combination outperformed other groups on both time points, highlighting its sustained immune-enhancing effects.

## 4. Discussion

The search for effective natural alternatives to in-feed antibiotics necessitates a deep understanding of how individual compounds and their combinations modulate key physiological processes like growth and immunity. This study provides a comprehensive evaluation of the individual and combined effects of sanguinarine (SA) and *Achyranthes bidentata* polysaccharides (ABPS) on yellow-feathered broilers using a robust factorial design. Our findings not only confirm the individual benefits of each additive but, more importantly, reveal a significant synergistic interaction that enhances both growth performance and immune function, offering a promising multi-faceted strategy for sustainable poultry production.

### 4.1. Interplay Between Growth Performance and Immune Modulation: A Stage-Dependent and Synergistic Relationship

A central finding of our study is the stage-dependent efficacy of SA and ABPS, and the emergent synergistic effects when they are combined. SA supplementation significantly improved ADG specifically during the critical starter phase (0–2 weeks), which aligns with previous studies showing that SA enhances early growth by improving nutrient digestibility and hepatic antioxidant capacity [11,27,28]. This early boost is crucial, as it establishes a strong foundation for subsequent growth. The mechanism may involve SA’s ability to reduce intestinal decarboxylation of aromatic amino acids and modulate the Trp–serotonin pathway, thereby stimulating feed intake [29]. In contrast, ABPS exerted its most pronounced effect on feed efficiency, significantly reducing FCR throughout the experiment, with an optimal dose of 500 mg/kg. This suggests that ABPS acts through a different mechanism, likely by improving intestinal mucosal morphology and modulating the cecal microbiome to enhance nutrient absorption [30].

This synergy implies that SA and ABPS target complementary physiological pathways—SA potentially optimizing digestive processes and ABPS enhancing gut health—which collectively support more robust and efficient growth over the entire production cycle, particularly during the later finisher phase (days 29–56). The most compelling evidence for an intertwined effect comes from the significant interaction between SA and ABPS on ADG and ADFI at 8 weeks of age, which marks the end of the production cycle. While individual additives showed stage-specific benefits, their combination led to significantly improved performance by the end of the finisher phase (day 56). This is visually supported by the significant effects of various combinations (e.g., A_2_B_2_, A_3_B_6_, A_4_B_4_) on 8-week performance metrics shown in Figure 5.

### 4.2. Synergistic Enhancement of Immune Organ Development and Function

The immune organ indices provide a clear physiological basis for the observed health benefits and their interconnection with growth. We found that both SA and ABPS individually enhanced the relative weights of the thymus, spleen, and bursa of Fabricius, which are primary indicators of immune system development [31,32]. The optimal doses for these effects were 0.7 mg/kg for SA and 500 mg/kg for ABPS, as illustrated in Figure 6 and Figure 7. The increase in immune organ weights suggests a systemic immunostimulatory effect, which is consistent with previous reports for ABPS in broilers [33] and aligns with the known capacity of plant polysaccharides to activate innate immune cells [16,17].

Crucially, a significant synergistic interaction was observed for thymus weight at 4 weeks (Figure 8). Combinations such as A_3_B_3_, A_4_B_6_, and A_5_B_5_ resulted in greater thymic development than would be expected from the sum of their individual effects. The thymus is a primary lymphoid organ responsible for T-cell maturation, and its enhanced development at an early age indicates a potentiation of cell-mediated immunity. This synergistic effect on the foundational architecture of the immune system likely underpins the enhanced functional responses seen in lymphocyte proliferation, creating a direct link between organ development and cellular function.

### 4.3. A_3_B_3_ Combination: A Synergistic Peak in Cell-Mediated Immunity Links Immunity to Growth

The lymphocyte proliferation assay serves as a direct functional measure of cell-mediated immune competence. Our results demonstrate that while both additives are effective individually, their combination yields a supra-additive effect. The most significant finding is the powerful and consistent synergy exhibited by the A_3_B_3_ combination (0.5 mg/kg SA + 300 mg/kg ABPS), which induced the highest lymphocyte proliferation at both 4 and 8 weeks. This is vividly captured in the heat map (Figure 10), where A_3_B_3_ stands out with the darkest red shading, indicating peak OD values.

### 4.4. Limitations and Future Perspectives

A limitation of this study is the primary focus on lymphocyte proliferation; future research should include measurements of antibody titers, cytokine profiles, and gut health parameters to provide a more comprehensive view of the immune response and the gut-immunity axis. Furthermore, elucidating the precise molecular mechanisms behind the SA-ABPS synergy, particularly concerning receptor binding and signal transduction in immune cells, would be a valuable next step.

## 5. Conclusions

In conclusion, our study moves beyond the characterization of individual effects to demonstrate a significant synergistic interaction between SA and ABPS. This synergy manifests in enhanced immune organ development, superior lymphocyte proliferation, and improved late-stage growth performance in yellow-feathered broilers. The combination of 0.5 mg/kg SA and 300 mg/kg ABPS (A_3_B_3_) is particularly effective for boosting cell-mediated immunity. These findings underscore that the future of antibiotic alternatives lies not in single compounds but in rationally designed combinations that target multiple physiological pathways synergistically. This work provides a strong scientific foundation for developing SA and ABPS as a core component of integrated, natural, and effective strategies for antibiotic-free poultry production.

## Figures and Tables

**Table 1:** The composition of the basal diet.

Item	Starter (0 to 28 Day)	Finisher (29 to 56 Day)
Ingredient, %		
Ground yellow maize	56.61	60.51
dehulled Soybean meal	36.00	32.00
Soybean oil	3.00	3.00
Di-calcium phosphate	1.80	1.90
Limestone	1.00	1.00
NaCl	0.30	0.30
DL-Met	0.14	0.14
Choline chloride	0.15	0.15
Premix ^1^	1.00	1.00
Nutrient level ^2^		
ME, MJ/kg	12.34	12.47
CP, %	21.19	19.64
Lys, %	1.07	0.96
Met, %	0.46	0.44
Cys, %	0.37	0.34
Ca, %	1.04	0.91
P, %	0.69	0.67

^1^ Supplied, per kilogram of diet: Cu, 10 mg; Fe, 90 mg; Mn, 90 mg; Zn, 50 mg; Se, 0.2 mg; I, 0.4 mg; Co, 0.4 mg; vitamin A, 5000 IU; cholecalciferol, 500 IU; vitamin E, 10 IU; riboflavin, 6.0 mg; pantothenic acid, 12 mg; niacin, 35 mg; cobalamin, 10 µg; biotin, 0.8 mg; folic acid,0.8 mg; thiamine, 1.5 mg; and pyridoxine, 1.5 mg. ^2^ Based on the Nutrient Requirements of yellow-feathered broilers (China, NY/T 33-2004) and the Nutrient Requirements of Broilers (NRC, 1994). DL-Met: DL-Methionine; ME: Metabolizable Energy; CP: Crude Protein.

**Table 2:** Dietary levels of SA and ABPS *.

SA mg/kg	ABPS mg/kg					
	B_1_ 0	B_2_ 200	B_3_ 300	B_4_ 400	B_5_ 500	B_6_ 600
A_1_ 0	A_1_B_1_	A_1_B_2_	A_1_B_3_	A_1_B_4_	A_1_B_5_	A_1_B_6_
A_2_ 0.4	A_2_B_1_	A_2_B_2_	A_2_B_3_	A_2_B_4_	A_2_B_5_	A_2_B_6_
A_3_ 0.5	A_3_B_1_	A_3_B_2_	A_3_B_3_	A_3_B_4_	A_3_B_5_	A_3_B_6_
A_4_ 0.6	A_4_B_1_	A_4_B_2_	A_4_B_3_	A_4_B_4_	A_4_B_5_	A_4_B_6_
A_5_ 0.7	A_5_B_1_	A_5_B_2_	A_5_B_3_	A_5_B_4_	A_5_B_5_	A_5_B_6_
A_6_ 0.75	A_6_B_1_	A_6_B_2_	A_6_B_3_	A_6_B_4_	A_6_B_5_	A_6_B_6_

* A 2-factorial arrangement of treatments was used for the trial. A factor was sanguinarine (SA), with six levels were set as A_1_ 0 mg/Kg, A_2_ 0.4 mg/Kg, A_3_ 0.5 mg/Kg, A_4_ 0.6 mg/Kg, and A_5_ 0.7 mg/Kg, A_6_ 0.75 mg/Kg; B factor was *Achyranthes bidentata* polysaccharides (ABPS), with six levels set as B_1_ 0 mg/Kg, B_2_ 200 mg/Kg, B_3_ 300 mg/Kg, B_4_ 400 mg/Kg, B_5_ 500 mg/Kg, and B_6_ 600 mg/Kg.

**Table 3:** Statistical analysis on performance of ABPS, SA and their combination in broilers.

Items	*p* Value		
	ABPS	SA	ABPS*SA
ADFI (g)			
0–2 wk	0.245	0.018	0.221
0–4 wk	0.194	0.15	0.064
0–6 wk	0.001	0.045	0.125
0–8 wk	0.838	0.037	0.03
FCR			
0–2 wk	<0.001	<0.001	0.71
0–4 wk	<0.001	0.021	0.351
0–6 wk	<0.001	<0.001	0.307
0–8 wk	<0.001	<0.001	0.223
ADG (g)			
0–2 wk	0.17	0.006	0.217
0–4 wk	0.27	0.123	0.063
0–6 wk	0.001	0.068	0.113
0–8 wk	0.85	0.052	0.002
Mortality (%)			
0–4 wk	0.036	0.07	1
0–8 wk	0.136	0.01	0.989

ADFI: average daily feed intake; ADG: average daily body weight gain; FCR: feed conversion ratio.

**Table 4:** Statistical analysis on lymphocyte proliferation of ABPS, SA and their combination in broilers.

Items	*p* Value		
	ABPS	SA	ABPS*SA
lymphocyte proliferation			
4 wk	<0.001	<0.001	<0.001
8 wk	<0.001	<0.001	<0.001

## Data Availability

The original contributions presented in this study are included in the article. Further inquiries can be directed to the corresponding authors.

## Abbreviations

SA: Sanguinarine. ABPS: *Achyranthes bidentata* polysaccharides. DL-Met: DL-Methionine. ME: Metabolizable Energy. CP: Crude Protein. ADFI: average daily feed intake. ADG: average daily body weight gain. FCR: feed conversion ratio.
